# Intermittent Pneumatic Compression as an Unrecognized Source of Neuromonitoring Oscillations

**DOI:** 10.1007/s10877-026-01423-1

**Published:** 2026-02-27

**Authors:** Ricard Valero, Miguel Medina, Alberto Abad, Javier Bellafont, Enrique Cepero

**Affiliations:** 1 Department of Anesthesiology and Resuscitation, Hospital Clinic de Barcelona, University of Barcelona, Institut d’Investigacions Biomédiques August Pi i Sunyer (IDIBAPS), Network Center for Biomedical Research in Mental Health (CIBERSAM), Barcelona, Spain; 2 Department of Anesthesiology, Hospital Clínic de Barcelona, University of Barcelona, Barcelona, Spain; 3 Anesthesiology of Hospital Metropolitano , Quito, Ecuador; 4 Department of Anesthesiology, Hospital Sant Joan of Reus, Tarragona, Spain; 5 RN. Surgical area, Hospital Clínic de Barcelona, University of Barcelona, Barcelona, Spain

**Keywords:** Intermittent Pneumatic Compression, Processed EEG artefact, Multimodal Neuromonitoring

**To the Editor**,

Multimodal neuromonitoring plays a pivotal role in preserving cerebral homeostasis during neurosurgical procedures. We report an unusual intraoperative observation in which the activation of an intermittent pneumatic compression (IPC) device produced synchronous, rhythmic fluctuations in both the bispectral index (BIS) and regional cerebral oxygen saturation (rSO₂).

A 42-year-old woman underwent elective craniotomy for resection of a right parietal cavernoma. Anesthesia was maintained using total intravenous anesthesia (TIVA) with propofol and remifentanil. During the procedure, a distinctive oscillatory pattern was observed simultaneously in the Density Spectral Array (DSA) derived from the BIS monitor (Covidien, Mansfield, MA, USA) and in rSO₂ values measured by near-infrared spectroscopy (Medtronic/Covidien, Mansfield, MA, USA). This pattern displayed a characteristic “sawtooth” morphology and coincided with cyclical increases in mean arterial pressure accompanied by reflex bradycardia (Fig. 1). Careful real-time observation confirmed that these changes were strictly phase-locked with the inflation–deflation cycles of the IPC device. Discontinuation of IPC resulted in immediate stabilization of all monitored parameters, while reactivation reproducibly restored the oscillatory pattern.

IPC devices function as a form of cyclic “auto-transfusion”, transiently increasing venous return and cardiac output (CO).[1, 2] This hemodynamic augmentation is known to enhance cerebral blood flow and may plausibly account for the observed increases in cerebral oxygenation. Supporting this mechanism, recent evidence demonstrates that IPC improves coupling between cerebral oxyhemoglobin concentrations and arterial blood pressure.[3] The concomitant oscillations observed in BIS values may reflect a manifestation of neurovascular coupling, whereby mechanically induced surges in cerebral perfusion modulate baseline cortical electrical activity.[4] Resting cerebral perfusion has been shown to correlate closely with EEG power, and individual alpha peak frequencies are associated with regional cerebral blood flow patterns.[5].

Alternatively, these changes may be mediated indirectly through alterations in propofol pharmacokinetics secondary to IPC-induced fluctuations in CO. Kurita and colleagues demonstrated that increases in CO can reduce plasma and effect-site propofol concentrations via a “washout” phenomenon, potentially leading to transient lightening of anesthetic depth.[6, 7] The accompanying autonomic response, manifested as reflex bradycardia, further underscores the integrity of baroreceptor-mediated cardiovascular control during these hemodynamic oscillations.[8].

Our observation is also consistent with a broader body of evidence showing that processed EEG (pEEG) indices, including BIS, are susceptible to a wide range of non-anesthetic influences.[9, 10] Mechanical, electrical, and physiological factors such as cardiopulmonary bypass, pacemakers, fluid-warming devices, electromyographic activity, altered cerebral perfusion, or electromagnetic interference from implanted or external devices have all been reported to produce misleading pEEG changes that do not reflect true hypnotic state. These artifacts may occur despite apparently adequate signal quality indicators and can mimic arousal or inadequate anesthesia, potentially prompting inappropriate therapeutic responses. Our case adds intermittent pneumatic compression to this growing list of conditions in which extracranial or systemic mechanisms may masquerade as cortical effects, reinforcing the need for cautious interpretation of pEEG values within their full physiological and technical context. We will remain attentive to the occurrence of additional cases, which may allow a more in-depth analysis and help better distinguish the underlying clinical mechanisms from monitoring-related artifacts.

**Fig. 1:**
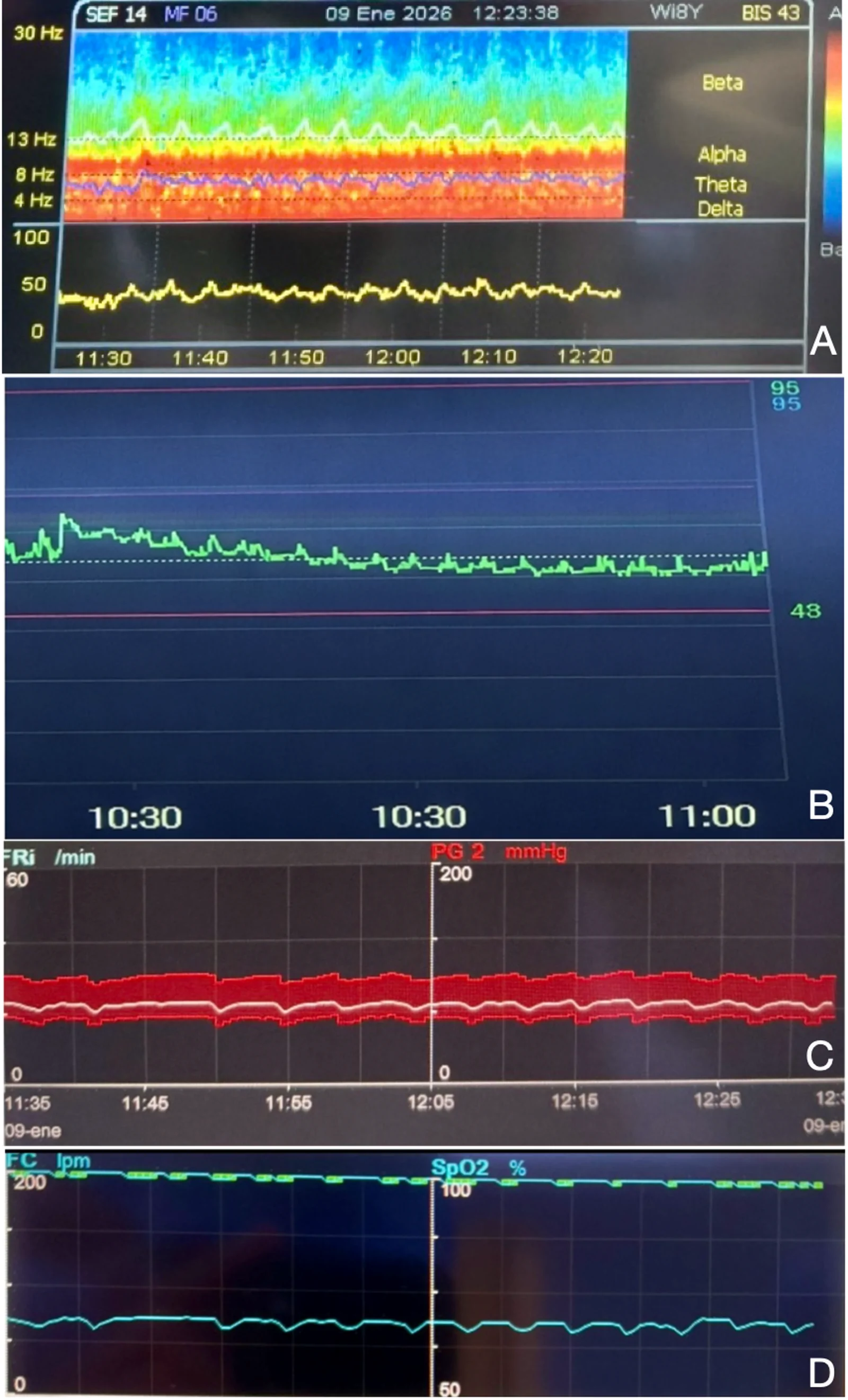
Multimodal Monitoring Trends During intermittent pneumatic compression (IPC) activation and stop: **(A)** BIS and Density Spectral Array showing IPC-synchronised oscillations. **(B)** Regional cerebral oxygen saturation (rSO₂). **(C)** Invasive arterial blood pressure. **(D)** Heart rate trends showing reflex bradycardia

## Data Availability

No datasets were generated or analysed during the current study.
